# Development of a Versatile Method to Construct Direct Electron Transfer-Type Enzyme Complexes Employing SpyCatcher/SpyTag System

**DOI:** 10.3390/ijms24031837

**Published:** 2023-01-17

**Authors:** Takumi Yanase, Junko Okuda-Shimazaki, Ryutaro Asano, Kazunori Ikebukuro, Koji Sode, Wakako Tsugawa

**Affiliations:** 1 Joint Department of Biomedical Engineering, The University of North Carolina at Chapel Hill and North Carolina State University, Chapel Hill, NC 27599, USA; 2 Department of Biotechnology and Life Science, Graduate School of Engineering, Tokyo University of Agriculture and Technology, 2-24-16 Naka-cho, Koganei 184-8588, Tokyo, Japan

**Keywords:** biosensors, direct electron transfer, SpyCatcher/SpyTag, glucose dehydrogenase, d-amino acid oxidase, l-lactate oxidase, biomedical engineering

## Abstract

The electrochemical enzyme sensors based on direct electron transfer (DET)-type oxidoreductase-based enzymes are ideal for continuous and in vivo monitoring. However, the number and types of DET-type oxidoreductases are limited. The aim of this research is the development of a versatile method to create a DET-type oxidoreductase complex based on the SpyCatcher/SpyTag technique by preparing SpyCatcher-fused heme *c* and SpyTag-fused non-DET-type oxidoreductases, and by the in vitro formation of DET-type oxidoreductase complexes. A heme *c* containing an electron transfer protein derived from *Rhizobium radiobacter* (CYTc) was selected to prepare SpyCatcher-fused heme *c*. Three non-DET-type oxidoreductases were selected as candidates for the SpyTag-fused enzyme: fungi-derived flavin adenine dinucleotide (FAD)-dependent glucose dehydrogenase (GDH), an engineered FAD-dependent d-amino acid oxidase (DAAOx), and an engineered FMN-dependent l-lactate oxidase (LOx). CYTc-SpyCatcher (CYTc-SC) and SpyTag-Enzymes (ST-GDH, ST-DAAOx, ST-LOx) were prepared as soluble molecules while maintaining their redox properties and catalytic activities, respectively. CYTc-SC/ST-Enzyme complexes were formed by mixing CYTc-SpyCatcher and SpyTag-Enzymes, and the complexes retained their original enzymatic activity. Remarkably, the heme domain served as an electron acceptor from complexed enzymes by intramolecular electron transfer; consequently, all constructed CYTc-SC/ST-Enzyme complexes showed DET ability to the electrode, demonstrating the versatility of this method.

## 1. Introduction

The principles of current electrochemical enzyme sensors are categorized into three generations [1,2]: the first generation utilizes oxygen as an electron acceptor, the second generation uses synthetic electron acceptors or mediators, and the third generation employs enzymes capable of direct electron transfer (DET). Enzyme sensors employing DET-type oxidoreductases do not require oxygen or any electron mediators and can be operated under a low oxidation potential, which is advantageous for minimizing the impact of electrochemically active ingredients. Furthermore, toxic synthetic mediators are not necessary for the electrochemical measurements, and errors due to variations in the concentration of oxygen in the biological samples are eliminated. Hence, DET-type oxidoreductase-based enzyme sensors are ideal for continuous and in vivo monitoring. However, the number and types of DET-type oxidoreductases, which are inherently able to transfer electrons from their redox cofactor to the electrode, are limited.

The currently reported DET-type oxidoreductases are composed of two domains or two subunits, a catalytic domain or a catalytic subunit and an electron transfer domain or an electron transfer subunit [2,3,4,5], which are necessary to exhibit the DET ability to the electrode. The electron transfer domain/subunit serves as the primary electron acceptor of the catalytic domain/subunit via inter-/intramolecular electron transfer. The redox center of the most common electron transfer domain/subunit is heme *b* or heme *c*, which serves as the electron donor not only for the external electron acceptors but also for the electrode. Therefore, oxidoreductases harboring an electron transfer domain/subunit are capable of DET with an electrode.

Cofactor binding-type oxidoreductases, such as flavin adenine dinucleotide (FAD)-dependent glucose oxidase (GOx), glucose dehydrogenase (GDH), and pyrroloquinoline quinone (PQQ) glucose dehydrogenase (PQQGDH) have been extensively utilized for bioelectrochemical devices with the combination of mediators [1,3]. Fungi-derived FADGDHs are considered as the most popular enzyme for the current electrochemical glucose sensor owing to their high substrate specificity and oxygen insensitivity [1]. Owing to these great advantages, fungi FADGDHs have been utilized as not only a glucose-sensing element, but also as the labeling enzyme for electrochemical immunosensors [6,7,8]. However, these cofactor binding-type oxidoreductases, such as GOx and fungi-derived FADGDHs do not have the DET ability, since their redox cofactor, FAD, is buried deeply in the protein molecules. Considering the fact that only the free FAD of GOx showed voltametric signals, whereas the bound FAD did not [9] and also a recent article claimed the suspicious DET ability of GOx [10,11], to achieve DET using GOx or fungal FADGDH, the use of conductive nanomaterials [12,13] and/or the modification of the redox species onto the enzyme [14,15,16,17,18,19,20,21,22,23] or electrode [24] would be mandatory. Therefore, to overcome the limited availability of DET-type oxidoreductases, there have been reported several attempts to demonstrate the electrochemical enzyme sensors without soluble electron mediators based on non-DET-type oxidoreductases. The chemical modifications of ferrocene derivatives or ruthenium complexes onto an enzyme to improve the electron transfer between the enzymes and the electrode have been reported extensively [19,20,21,22,23]. However, the modification of enzymes with mediators often requires relatively complex procedures. We have reported a simple method for the modification of a mediator onto FAD-dependent oxidoreductases utilizing amine-reactive phenazine ethosulfate (arPES) [14,15,16,17,18]. Mediator modification at the position proximate enough to the cofactor of the enzyme, thereby showing the electron transfer with the electrode, has been described as quasi-direct electron transfer (quasi-DET).

Furthermore, we have developed strategies and methods to create DET-type oxidoreductases from non-DET-type oxidoreductases by recombinantly constructing fusion proteins between non-DET-type oxidoreductases and heme-containing electron transfer proteins. The first report involved the creation of DET-type PQQGDH [25] by genetically fusing the PQQGDH structural gene with the electron transfer domain (heme *c*) derived from the DET-type PQQ-enzyme and quinohemoprotein ethanol dehydrogenase [26] to form quinohemoprotein glucose dehydrogenase (QH-GDH). As a result, QH-GDH showed the intramolecular electron transfer between PQQ and fused heme *c* without losing its catalytic efficiency. Consequently, the fusion of PQQGDH with heme *c* showed the DET ability to the electrode. Subsequently, DET-type oxidoreductases have been developed from non-DET-type fungi-derived FADGDH [27,28] and l-lactate oxidase (LOx) [29]. The genetic fusion between non-DET-type oxidoreductases and a heme-containing electron transfer domain or heme protein has been recognized as a general method to create DET-type enzymes from non-DET-type enzymes [30].

However, there are some inherent limitations, such as protein folding [31,32] and post-translational modification [33,34,35,36], since the methods used to create DET-type oxidoreductases by genetic fusion techniques are dependent on the properties of the recombinant protein expression abilities of the host cell. The solubility of the recombinantly prepared fusion molecules is difficult to predict. In particular, the expression of the fusion proteins of two different origins (eukaryotes and prokaryotes) and/or different localization (cytosol, membrane, or periplasmic space) may encounter an improper folding pathway as well as transportation and localization, which may result in the expression as inclusion bodies or incorrectly folded inactive molecules. Furthermore, an uncommon issue exists that is associated with the expression of electron transfer domains/subunits containing heme *c*, which is the requirement for a specific post-translational modification. The maturation of heme *c* in the *Escherichia coli* recombinant host strain requires the post-translational modification of heme *c*, encoded in *ccm* operon, after heme *c* containing an electron transfer domain/subunit is translocated through the membrane via the general secretory pathway including *sec*-type signal peptide-dependent machinery [33,34,35,36]. Alternative post-translational modification is also available, i.e., heme lyase derived from yeast cells. Heme lyase can be coexpressed in the cytosol; therefore, heme-*c*-containing protein molecules can mature in the cytosol of the bacterial host [37,38]. However, the heme *c* binding site should be in its N-terminal region to be recognized and matured by the heme lyase system [39]. These complex situations make it difficult to create a fusion protein composed of a catalytic domain with flavoprotein oxidoreductases and heme *c* containing electron transfer domains.

Therefore, the aim of this research was to develop a novel and versatile method to create DET-type oxidoreductases from non-DET-type oxidoreductases by avoiding the above-mentioned inherent issues in the use of fusion techniques. We focused on the in vitro protein complex formation method, that is, the Catcher/Tag technology, specifically the SpyCatcher/SpyTag system. The SpyCatcher/SpyTag system consists of a split protein, Catcher, and Tag peptide, which autonomously reconstitute the covalently bonded complex by mixing them together [40,41]. This system was originally derived from a split protein of the CnaB2 domain from *Streptococcus pyogenes*. They are composed of two domains: SpyCatcher, a small protein molecule; and SpyTag, a small peptide comprising 13 amino acids. By mixing each fused molecule harboring either SpyCatcher or SpyTag, the original CnaB2 domain could be autonomously reconstituted. Identification of the fast, site-specific, and stable covalently bound complex-forming ability without additional chemical procedures enabled us to develop a fabrication method for antibody–enzyme complexes for enzyme-linked immunosorbent assay (ELISA) and electrochemical biosensing applications [6,7,42]. Inspired by these successive former reports, we believe that in vitro complex formation technology would enable the creation of DET-type oxidoreductases.

**Scheme 1:**
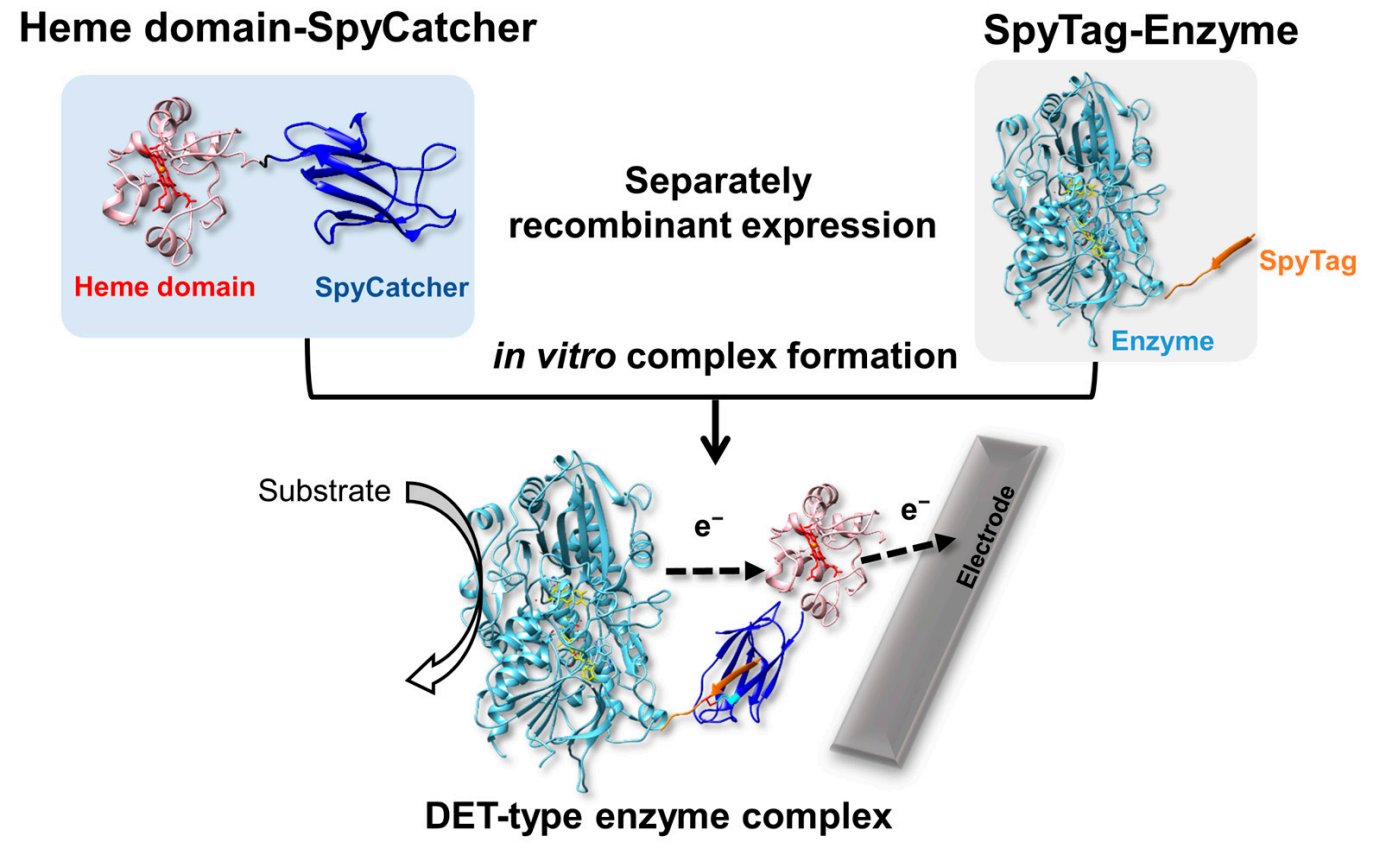
The architecture to create DET-type enzyme complex facilitated by SpyCatcher/SpyTag complex. In formed complex, heme *c* bound in electron transfer protein works as electron transfer domain from oxidoreductase to electrode with high proximity to cofactor of enzyme.

Herein, we report the development of a versatile method to create a DET-type oxidoreductase complex based on the SpyCatcher/SpyTag technique (Scheme 1) by preparing SpyCatcher-fused heme *c* and SpyTag-fused non-DET-type oxidoreductases, and by the in vitro formation of DET-type oxidoreductase complexes.

A heme *c* containing an electron transfer protein derived from *Rhizobium radiobacter* (CYTc) was selected. This molecule contained mono heme *c* covalently attached to the heme-binding motif and functioned as an electron acceptor of FAD-dependent glucoside 3-dehydrogenase [43]. Three non-DET-type oxidoreductases were selected as candidates for the SpyTag-fused enzyme as shown in Table 1: an engineered FAD-GDH, an engineered FAD-dependent D-amino acid oxidase (DAAOx), and an engineered FMN-dependent l-lactate oxidase (LOx) derived from *Aspergillus flavus* [44,45], *Rhodotorula gracilis* [18,46], and *Aerococcus viridans* [15,47], respectively.

CYTc-SpyCatcher (CYTc-SC) and SpyTag-Enzymes (ST-GDH, ST-DAAOx, ST-LOx), which contain flavin cofactors, were expressed separately. Both CYTc-SpyCatcher and each SpyTag-Enzyme were prepared as soluble molecules while maintaining their redox properties and catalytic activities, respectively. The CYTc-SC/ST-Enzyme complexes were formed by mixing the CYTc-SpyCatcher and SpyTag-Enzyme, and the complexes retained their original enzymatic activity. Remarkably, the heme domain served as an electron acceptor from complexed enzymes by intramolecular electron transfer; consequently, all constructed CYTc-SC/ST-Enzyme complexes showed DET ability to the electrode, demonstrating the versatility of this method.

## 2. Results

### 2.1. Recombinant Expression and Characterization of CYTc-SC

**Figure 1:**
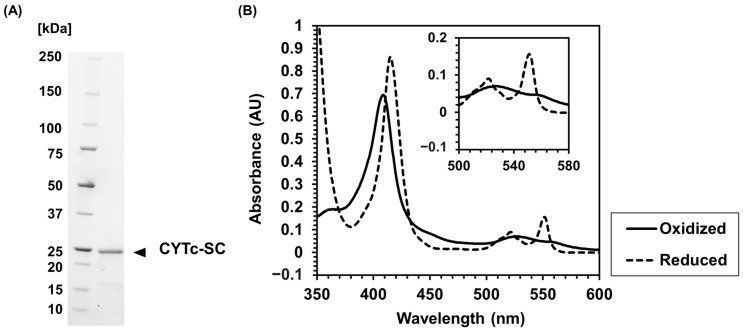
Characterization of recombinantly expressed CYTc-SpyCatcher (CYTc-SC). (**A**) SDS-PAGE analysis of the purified sample of CYTc-SC (expected molecular weight: 23 kDa). (**B**) Absorbance spectra in the range of 350–600 nm were recorded by a spectrometer. Solid and dashed lines indicate the spectrum of the purified sample of CYTc-SC in oxidative state treated with potassium ferricyanide and the spectrum of the purified sample of CYTc-SC in reduced state treated with sodium dithionite, respectively.

The expression vector for CYTc-SC (Supplementary Materials, Figure S1) was transformed into the *E. coli* BL21(DE3) host strain containing pEC86, which was inserted by *ccm* operon [36]. CYTc-SC was expressed as a soluble protein and purified using Ni^2+^ affinity chromatography. The SDS-PAGE analysis of purified CYTc-SC showed a band at the expected molecular weight of 23 kDa (Figure 1A). This result confirms the expression of CYTc-SC as a soluble fusion molecule in the host strain.

The redox properties of heme *c* in CYTc-SC were analyzed by investigating the absorbance spectra in the oxidized and reduced states (Figure 1B). Depending on its redox state, CYTc exhibits different absorption spectra, with two absorption peaks observed in the reduced state that are not observed in the oxidized state, which is typical for heme *c* [43]. The oxidized CYTc-SC showed a peak at 409 nm, corresponding to the Soret band. The reduced CYTc-SC showed a peak at 415 nm and two peaks at 521 nm and 551 nm corresponding to Q bands derived from the reduction of heme *c*. The absorbance spectra of oxidized/reduced CYTc-SC were similar to the previous observation with CYTc [43]. These results indicate that CYTc-SC was expressed with properly matured heme *c* with the original redox property upon fusion with SpyCatcher.

### 2.2. Complex Formation between CYTc-SC and ST-Enzymes

**Figure 2:**
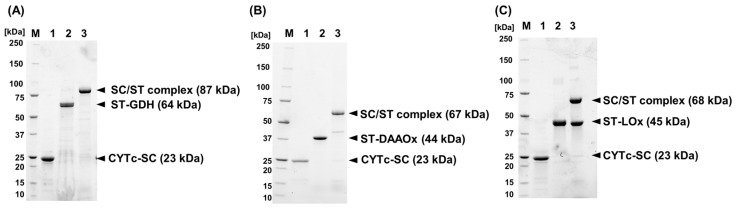
The evaluation of complex formation by SDS-PAGE analyses. (**A**) CYTc-SC/ST-GDH complex, (**B**) CYTc-SC/ST-DAAOx complex, and (**C**) CYTc-SC/ST-LOx complex. M, molecular weight marker; 1, CYTc-SC; 2, ST-Enzyme; and 3, mixed solution of CYTc-SC and each ST-Enzyme. All samples were purified by size-exclusion chromatography before SDS-PAGE analyses.

ST-GDH, ST-DAAOx, and ST-LOx were recombinantly expressed and purified using Ni^2+^ affinity chromatography. Each ST-Enzyme was prepared as a soluble molecule. Thus, the prepared samples were subjected to an enzyme analysis, which revealed that SpyTag-fusion did not have a significant negative impact on the selected enzymes (Table 2). To observe the complex formation between CYTc-SC/ST-Enzymes, both samples were mixed in an equivalent molar ratio and incubated on ice for 2 h. The complex formation was confirmed by band shifting using an SDS-PAGE analysis (Figure 2). By mixing CYTc-SC with ST-Enzymes, bands corresponding to the expected molecular weights were observed: 87 kDa (Figure 2A, lane 3) for CYTc-SC/ST-GDH, 67 kDa (Figure 2B, lane 3) for CYTc-SC/ST-DAAOx, and 68 kDa (Figure 2C, lane 3) for CYTc-SC/ST-LOx.

After the purification of each CYTc-SC/ST-Enzyme complex, the enzyme activities were measured, which revealed that the complex formation with CYTc-SC did not have a significant negative impact on enzyme activities. The determined *K*_M_ and *V*_max_ values of each ST-Enzymes or complexes shown in Table 2 and Supplementary Materials Figure S2 indicated that the complex-formed molecule majorly (>50%) showed dehydrogenase activity (CYTc-SC/ST-GDH: 2.0 U/µmol, CYTc-SC/ST-DAAOx: 0.10 U/µmol, CYTc-SC/ST-LOx: 1.4 U/µmol) compared to its original activity of the ST-Enzyme (ST-GDH: 3.2 U/µmol, ST-DAAOx: 0.22 U/µmol, ST-LOx: 2.0 U/µmol). These results indicate that the complex formation by the SpyCatcher/SpyTag reaction maintained the specific dehydrogenase activity of the CYTc-SC/ST-Enzyme complexes.

### 2.3. Investigation of Intramolecular Electron Transfer in CYTc-SC/ST-Enzyme Complexes

**Figure 3:**
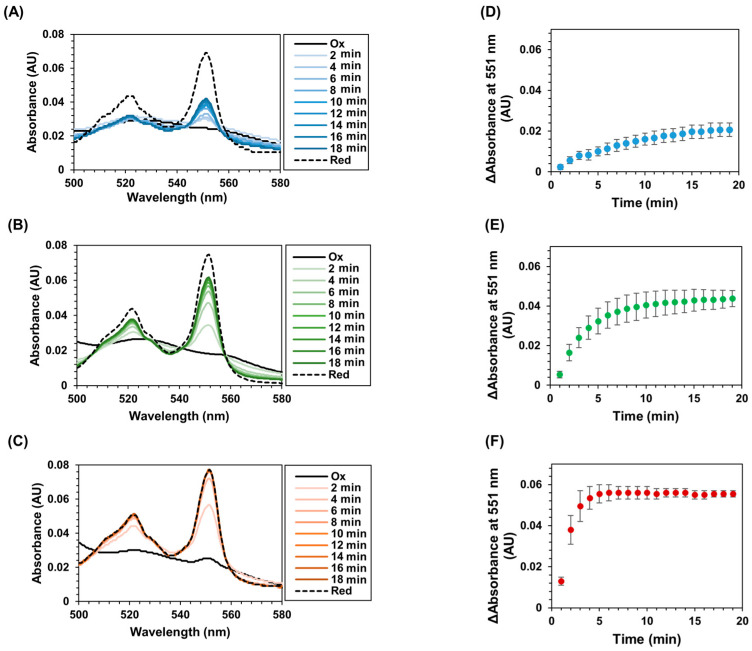
Time-dependent spectroscopic analyses of CYTc-SC/ST-Enzyme complexes. (**A**–**C**): Time-dependent absorbance spectrum changes of CYTc-SC/ST-Enzyme complexes in the presence of substrate. Absorbance spectra between 350 and 600 nm were recorded, and the range between 500 and 580 nm at every minute after the addition of substrate solution to the (**A**) CYTc-SC/ST-GDH, (**B**) CYTc-SC/ST-DAAOx, and (**C**) CYTc-SC/ST-LOx are shown. Black-solid line (Ox) and black-dashed line (Red) indicate each CYTc-SC/ST-Enzyme complex sample in oxidized state and reduced state treated with sodium dithionite, respectively. Recorded spectra in each minute were superimposed to observe time-dependent spectral change after the addition of substrate. Original recorded spectra between 350 and 600 nm are shown in Figure S3. (**A**–**C**): UV-vis spectrum analysis revealed the absorbance change at 551 nm of CYTc-SC/ST-Enzymes after addition of substrate. Absorbance spectra were measured every minute after the addition of substrate solution to the (**D**) CYTc-SC/ST-GDH, (**E**) CYTc-SC/ST-DAAOx, and (**F**) CYTc-SC/ST-LOx. The change in absorbance was calculated by subtracting the absorbance of the oxidized state from the absorbance recorded each minute. All experiments were performed in triplicate. Error bars represent the standard deviation for triplicated experiment.

To investigate the ability of intramolecular electron transfer from the flavin cofactor to heme *c* in the CYTc-SC/ST-Enzymes, changes in the absorbance spectra in the presence of the substrate were observed. The time-dependent absorbance spectra are shown in Figure 3 and Supplementary Materials Figure S3. After the addition of the substrate, a shift of the peak from 409 nm to 415 nm, corresponding to the Soret band of heme *c*, was observed. Simultaneously, an increase in the absorbance peaks at 521 nm and 551 nm derived from the Q bands of reduced heme *c* was observed in all cases of CYTc-SC/ST-Enzyme complexes. These results suggest that the addition of substrates for each complex resulted in the reduction of the flavin cofactor, followed by the intramolecular electron transfer from the reduced flavin cofactor to heme *c*. Consequently, the characteristically reduced heme *c* spectrum was observed. Thus, the CYTc-SC/ST-Enzyme complexes acquired the intramolecular electron transfer ability from flavin cofactor to heme with substrate oxidation by the complexed ST-Enzyme.

### 2.4. Electrochemical Evaluation of CYTc-SC/ST-Enzyme Complexes

**Figure 4:**
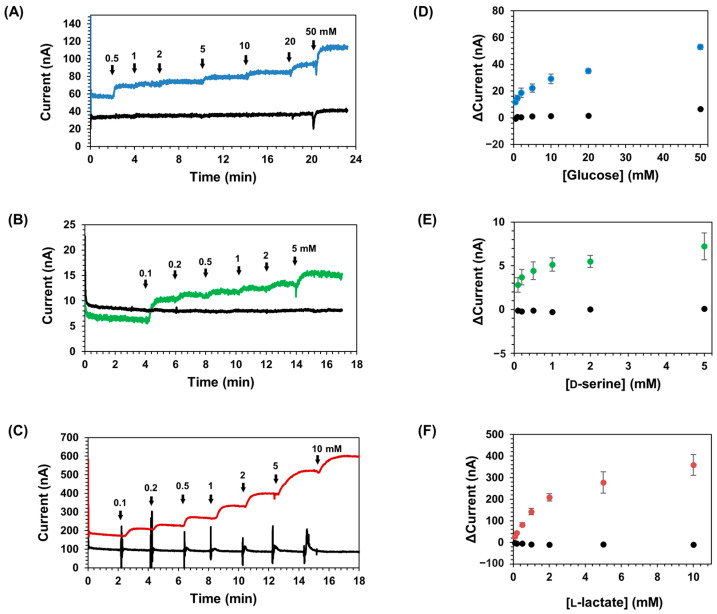
DET property analysis of CYTc-SC/ST-Enzyme complexes-immobilized electrodes by CA measurement. The response curves of electrodes immobilized (**A**) CYTc-SC/ST-GDH (blue line) and ST-GDH (black line), (**B**) CYTc-SC/ST-DAAOx (green line) and ST-DAAOx (black line), and (**C**) CYTc-SC/ST-LOx (orange and red lines) and ST-LOx (black line). The current response after the addition of each substrate was measured continuously by applying 400 mV vs. Ag/AgCl. Arrows indicate the time when substrate solution was added and the final substrate concentration in mM. The plot of measured current response change toward each substrate concentration for the electrodes immobilized (**D**) CYTc-SC/ST-GDH (blue circles) and ST-GDH (black circles), (**E**) CYTc-SC/ST-DAAOx (green circles) and ST-DAAOx (black circles), and (**F**) CYTc-SC/ST-LOx (red circles) and ST-LOx (black circles). All experiments were performed in triplicate. Error bars represent the standard deviation for triplicated experiments.

The DET ability of each CYTc-SC/ST-Enzyme was then investigated by chronoamperometry measurements using a CYTc-SC/ST-Enzyme complex-immobilized gold disk electrode. Figure 4 shows the representative current responses with the successive addition of the substrate: Figure 4A,D, CYTc-SC/ST-GDH-immobilized electrode; Figure 4B,E, CYTc-SC/ST-DAAOx-immobilized electrode; and Figure 4C,F, CYTc-SC/ST-LOx-immobilized electrode. These results revealed that the electrodes with CYTc-SC/ST-Enzyme complexes exhibited current increases in the sample addition, and the current increases were dependent upon the substrate concentration, even in the absence of an external electron acceptor. In contrast, the current response of each ST-Enzyme-immobilized electrode was hardly observed. The CYTc-SC/ST-GDH-immobilized electrode showed a current response with an apparent *I*_max_^app^ of 55 nA (Figure 4A,D, Table 3). Similarly, CYTc-SC/ST-DAAOx and CYTc-SC/ST-LOx showed a current response depending on the addition of the substrate with *I*_max_^app^ as 7.5 nA (Figure 4B,E, Table 3) or 410 nA (Figure 4C,F, Table 3). The current responses of CYTc-SC/ST-GDH- and CYTc-SC/ST-DAAOx-immobilized electrodes were saturated at a lower concentration according to their *K*_M_ values, which reflects the much lower *K*_M_^app^ values (5.6 mM for CYTc-SC/ST-GDH and 0.3 mM for CYTc-SC/ST-DAAOx; Table 3) compared with their *K*_M_ values (44 mM for CYTc-SC/ST-GDH and 1.9 mM for CYTc-SC/ST-DAAOx; Table 2). In the CYTc-SC/ST-LOx-immobilized electrode, *K*_M_^app^ from the current response was slightly lower than the *K*_M_ value (*K*_M_^app^ = 1.8 mM, *K*_M_ = 3.3 mM; Table 2 and Table 3). These results revealed that heme *c* in complexed CYTc-SC acted as an electron transfer domain from the flavin cofactor in ST-Enzyme to the electrode, achieving the DET between the enzyme and electrode. Thus, by preparing the SpyCatcher-fused heme *c* and SpyTag-fused non-DET-type oxidoreductases and by forming CYTc-SC/ST-Enzyme complexes in vitro, DET-type oxidoreductase complexes were successfully prepared.

## 3. Discussion

In this study, we demonstrated a novel and versatile method to create a DET-type enzyme complex based on an in vitro protein complex formation technique, which is an alternative to conventional gene fusion techniques. The formation of DET-type oxidoreductase complexes was confirmed by the observation of the intramolecular electron transfer between the flavin cofactor in ST-Enzyme and CYTc-SC and by the observation of the electron transfer from the CYTc-SC/ST-Enzyme to the electrode on the substrate addition. As we expected, our proposed method solved the addressed inherent issues in the preparation of DET-type oxidoreductases by genetic fusion techniques such as protein folding and post-translational modification. The in vitro complex formation between the heme protein and oxidoreductase by the SpyCatcher/SpyTag system waived the construction of the large fusion molecule between oxidoreductases and heme-containing electron transfer proteins. Instead, the fusion of the SpyTag peptide, which consists of only 13 amino acids, to oxidoreductases did not cause any significant impact on protein folding, and oxidoreductases with the fused SpyTag were produced as soluble proteins without a significant decrease in enzyme activities (Table 2). It was also demonstrated that the SpyCatcher-fused heme *c* molecule was also recombinantly produced as a soluble protein, and heme *c* was properly matured showing its characteristic two peaks at 521 nm and 551 nm, corresponding to the Q bands derived from the reduction of heme *c* (Figure 1). The in vitro complex formation between the ST-Enzyme and CYTc-SC was successively achieved with high yield for the CYTc-SC/ST-GDH complex and for the CYTc-SC/ST-DAAOx complex within 2 h (Figure 2). Since the formation of the CYTc-SC/ST-LOx complex was not completed in this period, further optimization for this case will be required. Consequently, this method enables us to create flavin-dependent oxidoreductases complexed with heme *c* in the bacterial host, which should be proceeded by different post-translational modifications from the one for the flavin enzymes.

The intramolecular electron transfer ability of each CYTc-SC/ST-Enzyme was confirmed by the observed increase in the Q bands of the reduced heme *c* when the enzyme substrate was added, which reduced the flavin. Since the catalytic activities of St-Enzymes and CYTc-SC/ST-Enzymes were confirmed (Table 2), the reduction of the flavin cofactor was expected to occur. Therefore, the increase in Q bands was due to the intramolecular electron transfer between flavin and heme *c* in CYTc-SC. A difference in the heme c reduction speed during the intramolecular electron transfer was observed, which depends on the oxidoreductases employed to prepare ST-Enzyme—CYTc-SC/ST-LOx > CYTc-SC/ST-DAAOx > CYTc-SC/ST-GDH (faster to slower). These results suggest that the intramolecular electron transfer from the flavin cofactor to heme *c* depends on the redox enzyme used for the complex formation with CYTc-SC. Furthermore, these trends are in good agreement with the DET abilities of CYTc-SC/ST-Enzymes, where CYTc-SC/ST-LOx showed the highest catalytic current.

Considering that all CYTc-SC/ST-Enzymes employed the same electron transfer protein, CYTc, the most crucial feature in the design of the complex would be the intramolecular electron transfer ability between ST-Enzyme and CYTc-SC. The efficiency of the electron transfer depended on the distance, orientation, and affinity/propulsion between ST-Enzyme and CYTc-SC. Another consideration was the quaternary structure of the oxidoreductases used in this study. While FAD-GDH is a monomeric enzyme, DAAOx is a homodimer, and LOx is a homotetramer in the C4 symmetrical structure. Considering that CYTc-SC/ST-LOx showed the highest and CYTc-SC/ST-DAAOx showed the second highest intramolecular electron transfer, the electron transfer between flavin and heme *c* must have occurred not only in the same “subunit”, but also by the inter-subunit electron transfer, which occurs between flavin and heme *c* in different subunits in homo-oligomers. Therefore, the observed increase in the Q bands is the sum of both the intra-subunit and inter-subunit electron transfers. Considering that the homotetrameric structure of LOx may provide more chances for inter-subunit electron transfer than homodimeric DAAOx and monomeric GDH, the quaternary structure of the original oxidoreductases will have a significant impact on the intramolecular electron transfer ability and, consequently, the DET ability of the CYTc-SC/ST-Enzymes.

To the best of our knowledge, this is the first report to achieve the internal electron transfer between the catalytic cofactor of oxidoreductase and the redox cofactor of an electron transfer protein in a complex formed by the SpyCatcher/SpyTag reaction. With respect to the design of DET-type enzymes, the ideal electron transfer domain should meet the requirements of small size and appropriate electrostatic surface charge to be suitable sterically and electrostatically as an electron acceptor to the catalytic domain or as an electron donor to the electrode. Indeed, our recent engineering approaches showed an enhanced DET ability of DET-type enzymes by downsizing the electron transfer subunit [48], or by mutating the amino acids at domain interface to suppress the electrostatic repulsion between the catalytic domain and electron transfer domain interfaces [49]. Additionally, the lower redox potential of the electron transfer domain was favorable for attaining a lower operating potential to avoid interference. The novel approach proposed in this study will provide a wide variety of design methods for DET-type enzyme molecules by selecting the heme *c* protein with respect to its size, redox potential, and electrostatic surface charge, regardless of the issues related to the post-translational modification of cofactors.

## 4. Materials and Methods

### 4.1. Chemicals and Materials

Sodium chloride (NaCl), dipotassium hydrogen phosphate, potassium dihydrogen phosphate (KH_2_PO_4_), imidazole, potassium ferricyanide, sodium dithionite, phenazine methosulfate (PMS), 2,6-dichlorophenolindophenol (DCIP), d(+)-glucose, d-serine, sodium l-lactate, sodium hydroxide (NaOH), sulfuric acid (H_2_SO_4_), 25% glutaraldehyde, and 1% (*w*/*v*) Nafion^TM^ were purchased from MilliporeSigma (Burlington, MA, USA). Mesoporous carbon particles (Cnovel P(4)050) were purchased from Toyo Tanso (Tokyo, Japan). All other chemicals were of reagent grade. Platinum wires were purchased from TANAKA KIKINZOKU (Tokyo, Japan). Gold disk (7 mm^2^) and silver/silver chloride (Ag/AgCl) reference electrodes were purchased from BAS Inc. (Tokyo, Japan).

### 4.2. Construction of the Expression Vectors

The amino acid sequences of the CYTc and SpyCatcher have been previously described [43,50]. To fuse the SpyCatcher to the C-terminus of CYTc, the following genes were synthesizedo: the structural gene of CYTc (residues 16–130) without its original signal sequence with an *Nco*I-*pelB* signal sequence HisTag at the 5′ end, and the structural gene of SpyCatcher (residues 21 to 104) with *Hin*dIII at the 3′ end was subsequently coded. This gene fragment, *Nco*I-*pelB*-HisTag-CYTc-SpyCatcher-*Hin*dIII, was amplified by a polymerase chain reaction (PCR) and inserted into pET30c(+) (Merck KGaA, Darmstadt, Germany) to construct an expression vector of the CYTc-SpyCatcher. To construct the ST-Enzyme expression vector, the structural gene of the SpyTag was added at the 5′ side of the structural gene of the FAD-dependent GDH derived from *Aspergillus flavus* [44,45], a G52V mutant of DAAOx derived from *Rhodotorula gracilis* [18,46], and an A96L/N212K double mutant of LOx derived from *Aerococcus viridans* [15,47] by PCR amplification. Each gene fragment coding, ST-GDH, ST-DAAOx, or ST-LOx was inserted to the *Nde*I-*Hin*dIII site of pET28a(+) (Merck KGaA, Darmstadt, Germany). The N-terminus of each ST-Enzyme contained a His-Tag derived from the vector. All the constructed expression vector information is shown in Supplementary Materials Figure S1.

### 4.3. Recombinant Expression

The CYTc-SpyCatcher expression vector was transformed into the host strain *E. coli* BL21(DE3) that transformed the accessory plasmid pEC86 [36], which expresses *ccmABCDEFGH* derived from *E. coli* for heme attachment to the heme *c*-binding motif (-CXXCH-) at the polypeptide chain in CYTc. Transformed cells were cultured in 500 mL baffled flasks containing 100 mL of ZYP-5052 medium (1% tryptone,0.5% yeast extract, 0.5% glycerol, 0.05% glucose, 0.2% lactose, 50 mM (NH_4_)_2_SO_4_, 50 mM KH_2_PO_4_, 50 mM Na_2_HPO_4_, and 1 mM MgSO_4_) [51], 50 µg/mL of kanamycin, and 50 µg/mL of chloramphenicol for 24 h at 100 rpm and 30 °C.

Each constructed expression vector for ST-Enzymes was transformed into the *E. coli* BL21(DE3) host strain. Transformed cells were cultured in 500 mL conical-shaped flasks containing 100 mL of ZYP-5052 medium. The culture conditions for ST-GDH, ST-DAAOx, and ST-LOx were 20 °C, 150 rpm, 24 h; 30 °C, 150 rpm, 24 h; and 30 °C, 150 rpm, 30 h, respectively, as described in a previous report [15,18,45].

The cells were collected, washed with 0.85% NaCl, and disrupted by ultrasonication in 20 mM potassium phosphate buffer (pH 7.0) containing 20 mM imidazole and 500 mM NaCl. Purification was performed using the following chromatographic steps. The supernatant of the disrupted cells was applied to a HisTrap HP column (Cytiva, Marlborough, MA, USA) equilibrated with 20 mM potassium phosphate buffer (pH 7.0) containing 20 mM imidazole and 500 mM NaCl. The adsorbed protein was eluted using a linear imidazole gradient (20–500 mM) in 20 mM potassium phosphate buffer (pH 7.0) containing 500 mM NaCl. Eluted proteins were dialyzed against the 20 mM potassium phosphate buffer (pH 7.0). The eluted fractions were analyzed using SDS-PAGE.

### 4.4. Preparation of Oxidized or Reduced Sample of CYTc-SC

To prepare the oxidized CYTc-SC sample, the purified sample (0.1 mM) was incubated with 1 mM potassium ferricyanide and then dialyzed against the 20 mM potassium phosphate buffer (pH 7.0). The reduction of CYTc-SC was investigated by adding excess sodium dithionite. Each sample was analyzed using a spectrometer to confirm the peaks derived from heme *c* and to confirm the redox properties of heme.

### 4.5. Formation of CYTc-SpyCatcher/SpyTag-Enzyme

To prepare the complex formed by the SpyCatcher/SpyTag system, 0.15 mM oxidized CYTc-SpyCatcher was mixed with 0.15 mM ST-GDH, ST-DAAOx, or ST-LOx and incubated on ice for 2 h. After incubation, each complex was purified by size-exclusion chromatography using Superdex 200 Increase 10/300 GL (Cytiva, Marlborough, MA, USA) with 20 mM potassium phosphate buffer (pH 7.0) containing 150 mM NaCl as the elution buffer to remove the unreacted molecules. Eluted samples were collected and analyzed by SDS-PAGE to determine the expected molecular weight of the SpyCatcher/SpyTag complex.

### 4.6. Enzyme Activity and Absorption Spectrum Analysis of the Complexes

The dye-mediated enzymatic activities of ST-Enzymes (ST-GDH, ST-DAAOx, and ST-LOx) and CYTc-SC/ST-Enzymes (CYTc-SC/ST-GDH, CYTc-SC/ST-DAAOx, and CYTc-SC/ST-LOx) were determined. The enzyme sample was incubated in a 20 mM potassium phosphate buffer (pH 7.0) containing 0.6 mM PMS, 0.06 mM DCIP, and various concentrations of substrate (glucose for GDH, d-serine for DAAOx, and l-lactate for LOx). The decrease in absorbance at 600 nm derived from the reduction of DCIP was monitored using a UV-1280 spectrometer (Shimadzu, Kyoto, Japan). In this assay, the reduction of 1 µmol of DCIP in 1 min was defined as 1 unit of dehydrogenase activity, which corresponded to the oxidation of 1 µmol of the substrate.

To observe the internal electron transfer from the flavin cofactor to heme *c* in each CYTc-SC/ST-Enzyme, the absorbance spectra of CYTc-SC/ST-Enzymes were measured using a UV-1800 spectrometer (Shimadzu, Kyoto, Japan). The enzyme solution was prepared in a 20 mM potassium phosphate buffer (pH 7.0). The substrate solution was then applied to the complex solution, and the absorbance spectra were recorded every 1 min for 20 min after the addition of the substrate.

### 4.7. Electrochemical Measurement

Gold disk electrodes (7 mm^2^) were polished with alumina slurry and electrochemically cleaned with 0.5 M NaOH and 0.5 M H_2_SO_4_, as described previously [52]. The enzyme ink was prepared by mixing 80 µg of mesoporous carbon particles (Cnovel P(4)050, Toyo Tanso, Tokyo, Japan) with ST-Enzyme solution (ST-GDH: 1 U, ST-DAAOx:0.1 U, ST-LOx: 1 U per electrode) or CYTc-SC/ST-Enzyme complex solution (CYTc-SC/ST-GDH: 1 U, CYTc-SC/ST-DAAOx: 0.1 U, CYTc-SC/ST-LOx: 1 U per electrode). Five microliters of each enzyme ink was dropped onto the cleaned Au electrode and dried. After drying, the ink-deposited electrode was cross-linked with 25% glutaraldehyde vapor for 30 min. The enzyme ink layer was then coated with 1 µL of 1% (*w*/*v*) Nafion solution in 20 mM potassium phosphate buffer and dried. The electrode was stored in 20 mM potassium phosphate buffer (pH 7.0) until use. The DET properties of the enzyme and complex immobilized electrode were evaluated by chronoamperometry measurements with an Ag/AgCl electrode and Pt wire as the reference electrode and counter electrode, respectively. Each electrode was immersed in 10 mL of 20 mM potassium phosphate buffer (pH 7.0) with continuous stirring. All chronoamperometric measurements were performed using a VSP Electrochemical Measurement System (Bio-Logic Science Instruments, Seyssinet-Pariset, France). A bias potential of +400 mV (vs. Ag/AgCl) was applied, and the current response was recorded using an electrochemical measurement system with the successive addition of the substrate solution. The output amperogram was analyzed using a band-reject filter with a Welch window. The rejected band frequencies ranged from 20 mHz to 50 mHz to remove the noise caused by continuous stirring. This smoothing analysis was performed using the EC-Lab software (Bio-Logic Science Instruments, Seyssinet-Pariset, France).

## 5. Conclusions

Owing to the benefits of bioelectrochemical applications and the low availability of DET-type enzymes in nature, oxidoreductases, which are capable of DET from the catalytic cofactor to the electrode, have been developed. Conventional methods for constructing DET-type enzymes are based on the recombinant expression of fusion proteins with an oxidoreductase and an electron transfer domain that harbors heme *b* or heme *c*. However, the application of this approach is sometimes restricted because of the issue related to the recombinant expression host, such as the difficulty of fusion protein expression as soluble and active molecules or the inherent problem with the post-translational modification pathway of cofactors. To overcome issues or limitations arising from the recombinant expression of fusion proteins, this study proposed an alternative and versatile method known as the in vitro protein complex formation technique to construct a DET-type enzyme complex facilitated by the SpyCatcher/SpyTag system.

Each CYTc-SC (as a heme *c* containing an electron transfer domain and ST-Enzymes as a catalytic domain) was separately prepared with its redox properties or enzymatic activities. CYTc-SC/ST-Enzyme complexes were formed by mixing, and the formed complex maintained its dehydrogenase activity. In addition, heme *c* in the complexed CYTc-SC functioned as an electron acceptor from the complexed enzyme via an internal electron transfer. Therefore, the CYTc-SC/ST-Enzyme complexes showed DET to the electrode compared to each ST-Enzyme alone. This novel method for creating DET-type enzyme complexes was demonstrated in three different oxidoreductases, indicating the versatility of this method.

## Figures and Tables

**Table 1:** Information about flavin-dependent oxidoreductases demonstrated in this research.

Oxidoreductase	Type	Cofactor	Origin	Dehydrogenase Activity (U/mg)	Dehydrogenase Activity (U/µmol)	Quaternary Structure	Molecular Weight of Monomer (kDa)	Reference
Glucose dehydrogenase V149C/G190C (GDH)	Dehydrogenase thermostable mutant	FAD	*Aspergillus flavus*	2.0 × 10^2^	5.6	Monomer	60	[44]
d-Amino acid oxidase G52V (DAAOx)	Oxygen-insensitive oxidase mutant	FAD	*Rhodotorula glacilis*	8.4	0.20	Homodimer	41	[18,46]
l-Lactate oxidase A96L/N212K (LOx)	Oxygen-insensitive oxidase mutant	FMN	*Aerococcus viridans*	1.7 × 10^2^	3.8	Homotetramer	44	[15]

**Table 2:** Dye-mediated dehydrogenase activities of CYTc-SC/ST-Enzyme complexes or ST-Enzymes.

		*K*_M_ (mM)	*V*_max_ (U/µmol)
GDH	ST-GDH	56	3.2
	CYTc-SC/ST-GDH	44	2.0
DAAOx	ST-DAAOx	2.6	0.22
	CYTc-SC/ST-DAAOx	1.9	0.10
LOx	ST-LOx	3.1	2.0
	CYTc-SC/ST-LOx	3.3	1.4

**Table 3:** Apparent *K*_M_ value (*K*_M_^app^) and apparent *I*_max_^app^ value calculated from DET current response.

	*K*_M_^app^ (mM)	*I*_max_^app^ (nA)
CYTc-SC/ST-GDH	5.6	55
CYTc-SC/ST-DAAOx	0.3	7.5
CYTc-SC/ST-LOx	1.8	410

## Data Availability

Any data or material that support the findings of this study can be made available by the corresponding authors upon request.

## Supplementary Materials

The following supporting information can be downloaded at: https://www.mdpi.com/article/10.3390/ijms24031837/s1.
