# Supplementary material for: Development of a Versatile Method to Construct Direct Electron Transfer-Type Enzyme Complexes Employing SpyCatcher/SpyTag System
Source: Int J Mol Sci. 2023 Jan 17;24(3):1837. doi: 10.3390/ijms24031837 (PMC9915066; doi:10.3390/ijms24031837)
Supplement: Supplementary file 1 [file ijms-24-01837-s001.zip › ijms-2154796-supplementary.pdf]

Supplementary Materials

Development of a Versatile Method to Construct Direct Electron Transfer-Type Enzyme Complexes Employing SpyCatcher/SpyTag System

Takumi Yanase[1,2,†], Junko Okuda-Shimazaki[1,2,†], Ryutaro Asano[2], Kazunori Ikebukuro[2], Koji Sode[1*], Wakako Tsugawa[2*]

[1] Joint Department of Biomedical Engineering, The University of North Carolina at Chapel Hill and North Carolina State University, Chapel Hill, NC 27599, USA

[2] Department of Biotechnology and Life Science, Graduate School of Engineering, Tokyo University of Agriculture and Technology, 2-24-16 Naka-cho, Koganei, Tokyo 184-8588, Japan

* Correspondence: ksode@email.unc.edu (K.S.); tsugawa@cc.tuat.ac.jp (W.T.); Tel.: +81-919-966-3550 (K.S.); +81-42-388-7789 (W.T.)

† These authors contributed equally to this work.

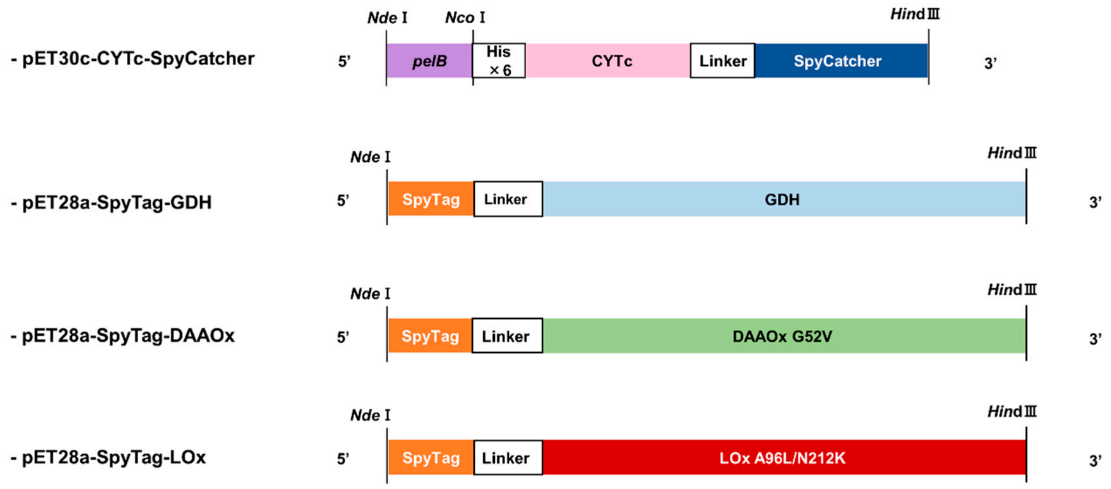

Figure S1. Inserted sequence of constructed expression vectors (pET30c-CYTc-SpyCatcher, pET28a-SpyTag-GDH, pET28a-SpyTag-LOx, and pET28a-SpyTag-DAAOx)
All linker regions were composed of 3 amino acids (-Gly-Thr-Gly-).

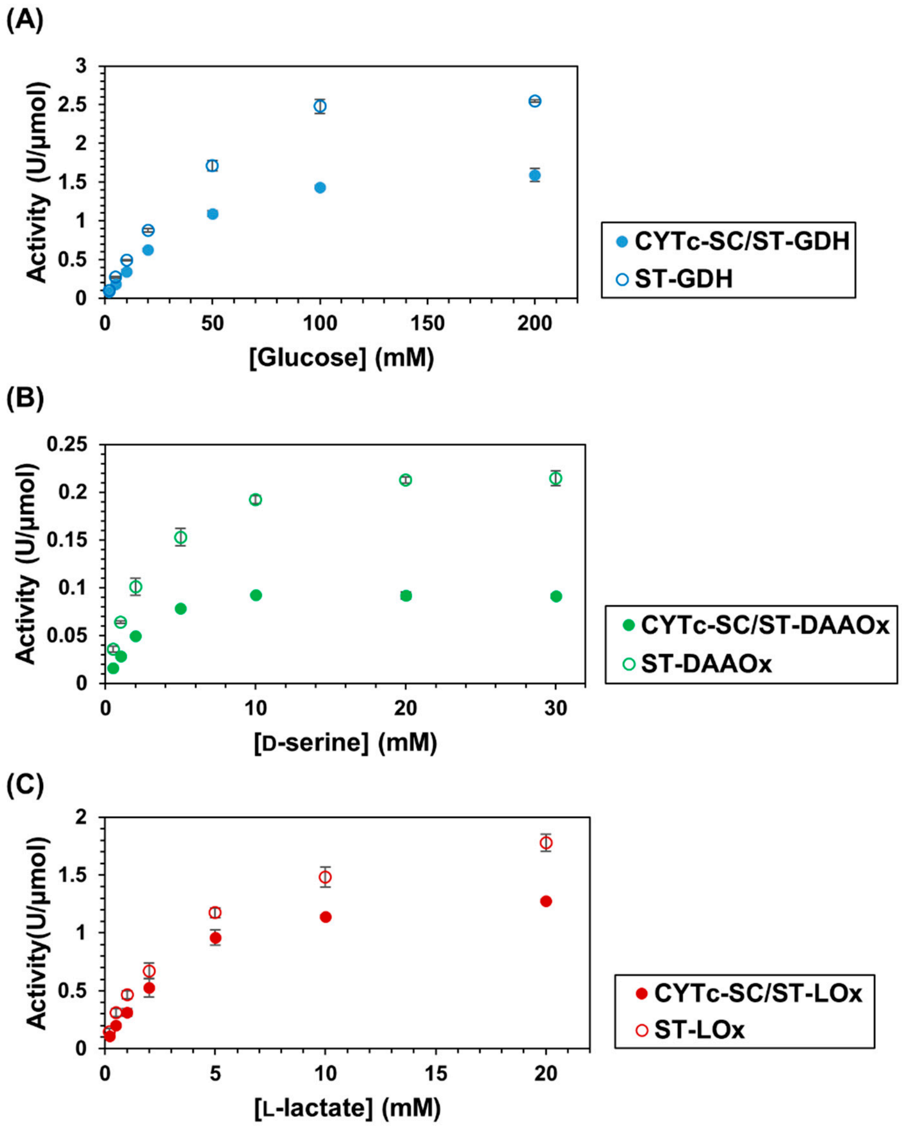

Figure S2. Dehydrogenase activity of each enzyme complex

The specific dehydrogenase activity of CYTc-SC/ST-Enzyme complexes or ST-Enzymes toward each substrate concentration was plotted as (A) CYTc-SC/ST-GDH, (B) CYTc-SC/ST-DAAOx, and (C) CYTc-SC/ST-LOx. Each dehydrogenase activity was determined using PMS/DCIP as the primary electron acceptor and redox dye. All experiments were performed in triplicate. Each error bars represents the standard deviation for triplicated experiment.

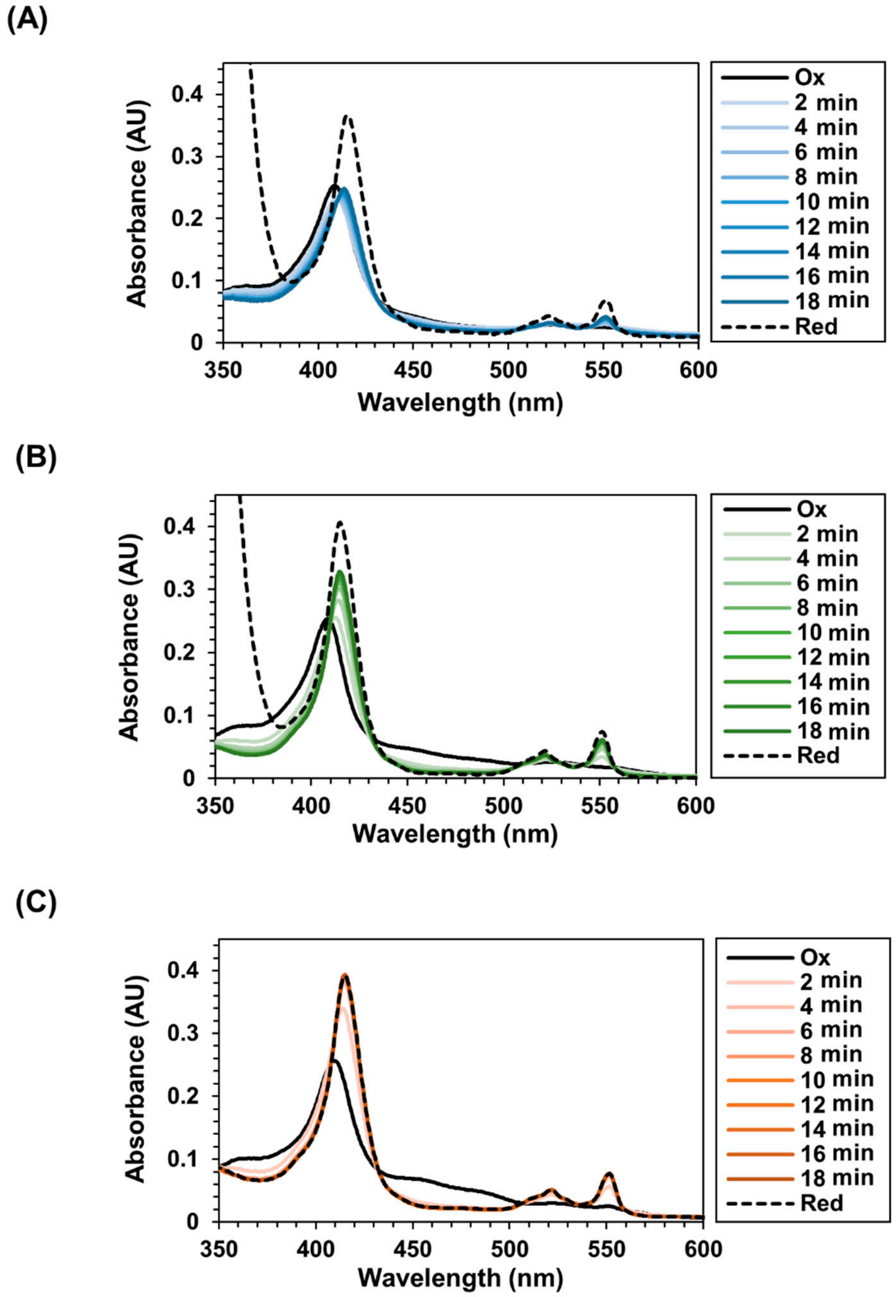

Figure S3. Time-dependent spectroscopic analysis of CYTc-SC/ST-Enzyme complexes

Time-dependent absorbance spectrum changes of CYTc-SC/ST-Enzyme complexes in the presence of substrate. Absorbance spectra between 350 and 600 nm were recorded, and at every minute after the addition of substrate solution to the (A) CYTc-SC/ST-GDH, (B) CYTc-SC/ST-DAAOx, and (C) CYTc-SC/ST-LOx are shown. Black-solid line (Ox) and black-dashed line (Red) indicate each CYTc-SC/ST Enzyme complex sample in oxidized state and reduced state treated with sodium dithionite. Recorded spectrum in each minute was superimposed to observe time-dependent spectral change after the addition of substrate.
